# Development and applications of a new neutron single-crystal diffractometer based on a two-dimensional large-area curved position-sensitive detector

**DOI:** 10.1107/S002188981300681X

**Published:** 2013-05-04

**Authors:** Chang-Hee Lee, Yukio Noda, Yoshihisa Ishikawa, Shin Ae Kim, Myungkook Moon, Hiroyuki Kimura, Masashi Watanabe, Yuki Dohi

**Affiliations:** a Neutron Science Division, Korea Atomic Energy Research Institute, Daejeon, Republic of Korea; b Institute of Multidisciplinary Research for Advanced Materials, Tohoku University, Sendai, Japan; c Institute of Materials Structure Science, KEK, Tokai, Japan; d New Industry Creation Hatchery Center, Tohoku University, Sendai, Japan

**Keywords:** single-crystal neutron diffraction, neutron diffractometers, curved two-dimensional position-sensitive detectors

## Abstract

A new single-crystal neutron diffractometer based on a large-area curved two-dimensional position-sensitive detector (C-2DPSD) has been developed. The diffractometer commissioning is almost complete, together with development of the measurement methodology and the raw data processing software package, the *Reciprocal Analyzer*, and the instrument is now ready to be launched for users. Position decoding of the C-2DPSD is *via* a delay-line readout method with an effective angular range of 110 × 54° in the horizontal and vertical directions, respectively, with a nominal radius of curvature of 530 mm. The diffractometer is equipped with a Ge(311) mosaic monochromator and two supermirror vacuum guide paths, one before and one after the monochromator position. The commissioning incorporates corrections and calibration of the instrument using an NaCl crystal, various applications such as crystallographic and magnetic structure measurements, a crystallinity check on large crystals, and a study on the composition or dopant content of a mixed crystal of (Tm_*x*_Yb_1−*x*_)Mn_2_O_5_. The installation of the diffractometer and the measurement method, the calibration procedure and results, the raw data treatment and visualization, and several applications using the large C-2DPSD-based diffractometer are reported.

## Abstract 2

A new reactor-based neutron single-crystal diffractometer was developed based on a large-area curved position-sensitive detector with a delay-line readout method. The instrumentation details are presented, including diffractometer construction, measurement method, raw data treatment and calibration, and various applications for structural studies are given to exploit the strengths of the diffractometer.

## 1. Introduction   

Single-crystal neutron diffraction has been used as a powerful technique for crystallographic and magnetic structure studies for more than half a century. Though several high-flux research reactors have become available in the past few decades around the world, the method still has several drawbacks, including the need for much larger single-crystal specimen volumes, usually ∼10 mm^3^ at present, than are required by X-ray analysis and the low data throughput, and there is an ever increasing demand for much higher neutron fluxes for sophisticated tasks. Roughly speaking, the time needed for typical laboratory X-ray single-crystal diffraction (SCD) on a specimen of ∼0.1 mm^3^ volume would be about three–four days at one temperature point, and for synchrotron X-ray SCD it is usually about one day for a ∼10 µm sample. In contrast to the case of X-ray diffraction, modern research-reactor-based neutron SCD takes from around three days to three weeks for data acquisition for a typical specimen size of ∼10 mm^3^. These days some reactor-based Laue diffractometers already take typically one day for 0.1 mm^3^ sample volumes for smaller unit-cell crystals, and newly built SCD machines at spallation neutron sources are targeted at approximately one day for 0.1 mm^3^ sample volumes, which is similar to the time required for the laboratory X-ray case.

In X-ray SCD, area detectors such as the imaging plate (IP) and charge-coupled device (CCD) have widely prevailed. On the other hand, use of these area detectors has been relatively rare in neutron SCD. There are position-sensitive detector (PSD)-type neutron single-crystal diffractometers such as BIX-3 (Tanaka *et al.*, 2002 ▶) and BIX-4 (Kurihara *et al.*, 2004 ▶) at JAEA, LADI (Cipriani *et al.*, 1995 ▶) and VIVALDI (Wilkinson *et al.*, 2002 ▶; McIntyre *et al.*, 2006 ▶) at ILL, and KOALA at ANSTO with an image plate. There are also SXD (Keen *et al.*, 2006 ▶) with a scintillator, CYCLOPS (Hewat, 2006 ▶) with a CCD, and a gas-counter-type PSD but with a different readout method (PCS; Kovalevsky *et al.*, 2010 ▶) at LANL, WOMBAT (Studer *et al.*, 2006 ▶) at ANSTO and D19 (Forsyth *et al.*, 2001 ▶; http://www.ill.eu/instruments-support/instruments-groups/instruments/d19/description/instrument-layout/) at ILL. Most of these machines target a broad range of biological or protein structural studies (Blakeley, 2009 ▶).

We have developed a new neutron single-crystal diffractometer based on a large-area curved two-dimensional position-sensitive neutron detector (Moon *et al.*, 2007 ▶, 2013 ▶), located at the research reactor HANARO. Our objective was to achieve a one–three day measurement time for an approximately 1 mm^3^ sample volume, as well as much faster measurements over a wider range of reciprocal space, even for large-unit-cell systems with unit-cell volumes of up to 30 000 Å^3^, thus reducing the total measurement time to within the practically available beam time.

The advantages of the IP method include a very large solid angle coverage under a pseudo-Laue or monochromatic beam, by a relatively low cost solution, using film-like technology with a high spatial resolution of ∼200 µm and a large dynamic range of ∼10^6^. On the other hand, this method is relatively vulnerable to the γ-ray background, has a lower signal-to-noise ratio and, the greatest disadvantage, has a slow readout time of the order of minutes. The gas-counter PSD-type diffractometer is superior to the image plate in terms of its high signal-to-noise ratio, which results from its differential detection characteristics, and very low γ-ray background, and the capability to be equipped with diverse sample environments. Although the local and global maximum count rates for the gas-counter-type PSD are relatively low, reactor-based neutron SCD signals fall within the range covered by such a detector; the lack of a wide solid angle coverage would be the only remaining problem.

**Figure 1:**
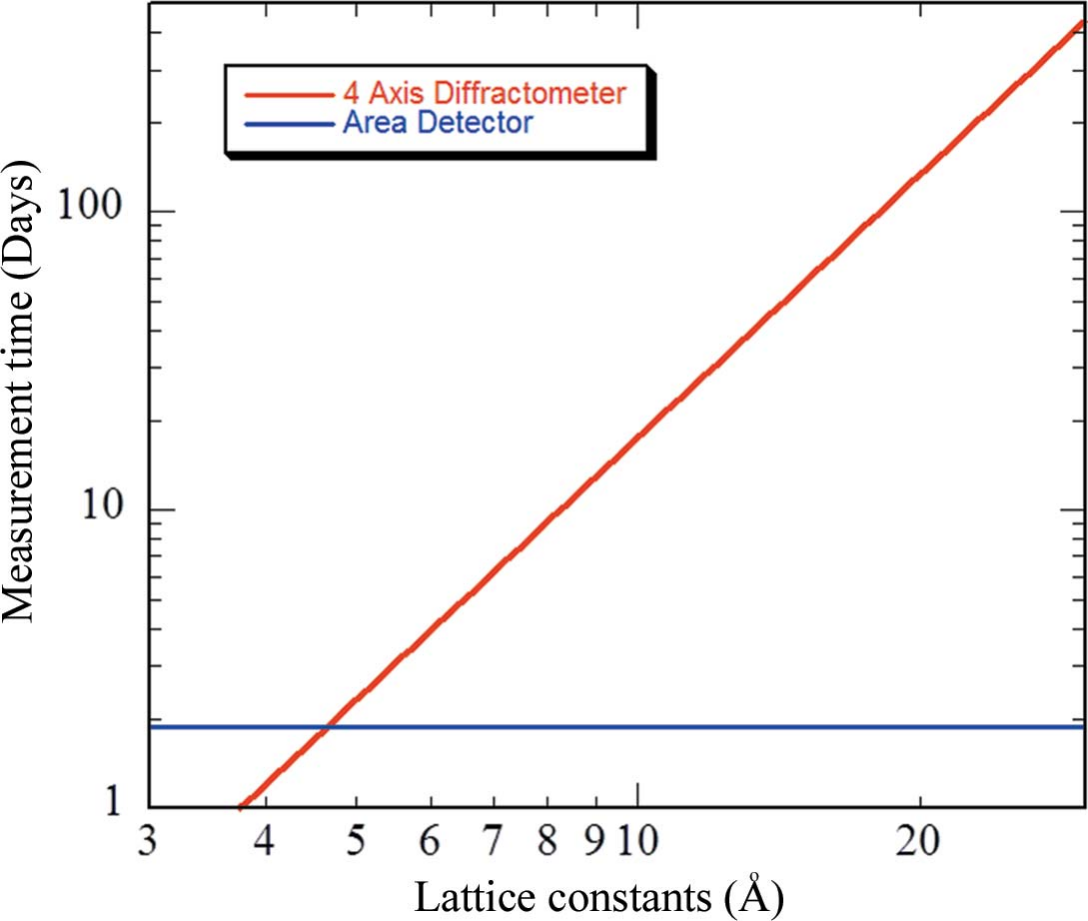
Comparison of measuring methods between a four-circle diffractometer and an area-detector-based diffractometer. Both axes are in logarithmic scale.

Fig. 1 ▶ shows a comparison of the throughput between a four-circle diffractometer (FCD) with a point detector and a diffractometer with an area detector. The model calculation was done with the assumption that the crystal system was orthorhombic, and intensity measurement by the FCD took ∼10 min per diffraction point using a 1.24 Å neutron beam and 2θ_max_ = 150°; for the area-detector case, a scan speed of 0.2° min^−1^ over a 180° range, covering a range of 2θ_max_ = 150°, χ_max_ = 45°, was used. We simply considered the measurement time for the number of reciprocal space points and neglected here the counting statistics and weaker intensity due to the larger unit-cell volume. It should be noted that the horizontal and vertical axes have a logarithmic scale. As the lattice constants become larger, there is a crossing point between the two measurement methods, and the measurement time by the area-detector method is stable over a wide range of lattice constants. It is evident that the area-detector method is superior for larger-unit-cell or low-symmetry systems. This also suggests that the area-detector method is quite efficient for searching a wide reciprocal space volume with no assumptions on the specimen under measurement.

Our development strategy was based on a conventional four-circle neutron single-crystal diffractometer with a tube detector, through a relatively small area, a flat two-dimensional detector, and finally a new dedicated diffractometer equipped with a large-area curved two-dimensional position-sensitive detector (C-2DPSD). The measurement methodology and raw data processing software were developed concurrently. Just after installation of the diffractometer, the commissioning work was done, incorporating corrections and calibration along with various applications such as chemical and magnetic structure measurements, large-crystal crystallinity checks, and a study on the composition or dopant content of a mixed crystal system.

In this report, the diffractometer installation and measurement method, calibration procedure and results, raw data treatment and visualization, and several applications using the large-area C-2DPSD-based diffractometer are presented.

## 2. Instrumentation   

### 2.1. Diffractometer components and monochromator   

The new diffractometer was installed in the ST3 beam port of HANARO; this beam port is shared by three different diffractometers. The first from the beam port end position is a high-intensity powder diffractometer having a bent perfect Si(511) slab as a monochromator. The second is the C-2DPSD-based diffractometer reported here, and the third is a BIX-type diffractometer with a bent perfect Si(111) monochromator. There had been only one instrument in this port, a neutron reflectometer, for 10 years from 2001. After this old instrument was relocated into the cold neutron guide hall in late 2010, the monochromator shield was modified and reinforced to accommodate three separate instruments by dividing and sharing the cross section of the beam port and different wavelengths in the spectrum.

**Figure 2:**
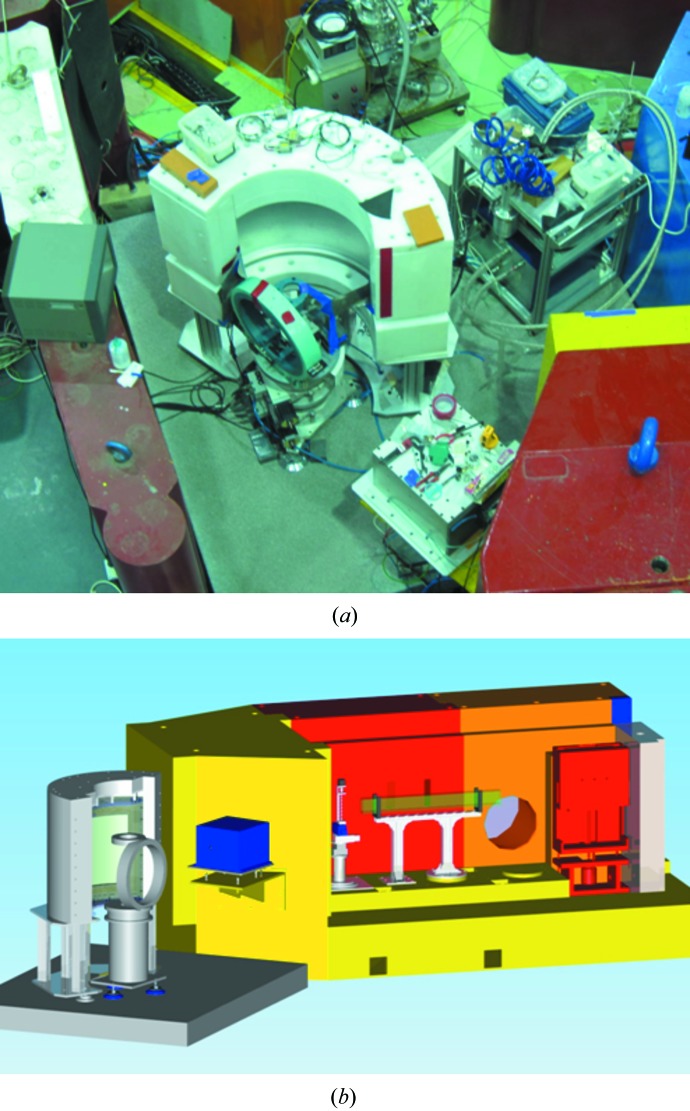
The diffractometer installed in the ST3 beam port at HANARO. (*a*) The photograph shows the installed configuration of the diffractometer. The customized sample vacuum chambers are not shown in the photograph. (*b*) The schematic shows the supermirror guide path in front of the monochromator (the rear guide path after the monochromator is not shown), two shutters (red and blue boxes) and the spectrometer with the detector shield.

Fig. 2 ▶(*a*) shows the arrangement of the diffractometer installed on the right side behind the monochromator shield of the ST3 beam port, and the schematic in Fig. 2 ▶(*b*) shows the internal configuration of the supermirror guide path in front of the monochromator (another supermirror inside the shield, positioned between the monochromator and the sample position, is not shown), the two shutters (shown as red and blue boxes) and the spectrometer with the detector shield.

The C-2DPSD has an active area of 937 × 520 mm, composed of single segmented wire frames with delay-line components on the cathode frames of wires (*X*) and strips (*Y*); the total delay time in the **X** direction (curved) is 240 ns, and that in **Y** (vertical) is 170 ns. The position decoding is done by an in-house-developed fast time-to-digital converter (TDC) with a minimum time resolution of 128 ps, which has four reading resolutions of 256, 512, 1024 and 2048 channels. Most of the time, the 512 channel mode is selected, and then the active area of 937 mm in **X** and 520 mm in **Y** results in 400 × 252 channels in the **X** and **Y** directions, respectively. The angular coverage of the detector, with a radius of 530 mm, is 110° in **X** and 54° in **Y**. The detector has a gas mixture of 3.9 atm of ^3^He and 0.7 atm of CF_4_ (1 atm ≃ 101.323 kPa) with a 3.4 cm detection depth, and the detection efficiency was obtained as ∼35% at 1.153 Å and 44% at 2.204 Å, with position resolutions in terms of the FWHM of a point spread function of 3.16 channels (6.4 mm) in **X** and 2.66 channels (4.8 mm) in the **Y** direction.

The diffractometer has a similar mechanical structure to a four-circle diffractometer, having an Eulerian cradle, but has a large-area curved detector instead of a point detector. It can use a Ge(311) mosaic crystal slab (with 15′ mosaicity) as primary monochromator to produce 1.153 Å radiation at a take-off angle of 39.5°, and because the slab has [311] as the surface normal and [0
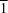
1] as the vertical direction, several other wavelengths, namely 0.7804, 2.204 and 0.7358 Å, are easily selected by rotating the slab, as summarized in Table 1 ▶ (Miyake *et al.*, 2010 ▶). The 2.204 Å radiation is important for measurements of large-unit-cell materials. The resolution function curve of the diffractometer in terms of the FWHM of peak profiles in 2θ is less than 0.5° in the 2θ range less than 110°. The 1.153 Å flux from the Ge(311) monochromator at the sample position was estimated by the Au-foil neutron activation analysis method to be ∼1.88 × 10^6^ neu­trons cm^−2^ s^−1^ at 27 MW thermal power. The 2.204 and 0.7804 Å fluxes were ∼60 and ∼30%, respectively, of the flux at 1.153 Å. The thickness of the installed Ge mosaic monochromator is 4 mm at present, but it will be replaced by a new Ge(311) mosaic monochromator with 6 mm thickness and improved mosaicity in the near future.

All the spectrometer parts and the area-detector shield unit can be moved by an air bearing method on a wide-area monolithic granite floor; the detector unit is actually floating at all times during operation, both to protect the detector against any mechanical vibration or shock and to allow it to be moved precisely and quickly.

The control and data acquisition system is configured on a PC running Linux, which controls the TDC-based C-2DPSD nuclear counting modules, a motion control unit and sample environment temperature controllers. All of the devices are controlled by the instrument control program *SPEC* (http://www.certif.com/content/spec/) on the Linux operating system. The TDC module was developed under collaboration with the Korea Astronomy and Space Science Institute (Nam *et al.*, 2007 ▶), and its dedicated device driver was also developed with *SPEC*.

The diffractometer has a customized room-temperature sample chamber and a 10 K closed-cycle refrigerator-type low-temperature sample chamber to reduce background from air scattering along the direct-beam direction over the wide opening of the area detector. The room-temperature chamber is of 220 mm-diameter cylinder shape with a magnetic seal coupling in the bottom plate, to accommodate fixed-beam-path inlet and outlet tubes attached to the chamber along the direct-beam direction. A cylinder-type Al vacuum shroud for the 10 K low-temperature cryostat was made, with 190 mm diameter and 2 mm thickness in the detector direction; the shroud has shielded inlet and outlet tubes of 25 mm diameter with a thin aluminium window. A magnetic seal coupling with 70 mm-diameter bore was applied to rotate the sample axis to keep the vacuum shroud fixed; a 1 mm-thickness Al thermal insulation and 50 µm-thickness Al sample can of 53 mm diameter were also made.

### 2.2. Measurement methods   

The measurement method using the area detector can be explained starting from the method of the conventional four-circle diffractometer, in which there are four angles (2θ, ω, χ, ϕ) describing the crystal orientation and diffraction condition. With a precisely known orientation matrix (UB matrix), each reflection, *i.e.* the reciprocal lattice point, is positioned with respect to the diffraction point in the scattering plane by successive rotations of the sample by the three angles (ω, χ, ϕ). Then the remaining parameter, 2θ, satisfies the diffraction condition of the scattering vector. Although the single-crystal diffraction points are treated as points, in reality, the reflections have finite dimensions depending on the crystal size and quality, *i.e.* mosaicity, and on the finite dimensions, angular divergence and wavelength dispersion of the beam.

In X-ray diffraction, IP- and CCD-based measurements dominate under the usual environments of high-intensity X-ray sources, such as a rotating-anode generator or synchrotron X-rays, and strong diffraction signals. For neutron diffraction, usually the flux to the sample is relatively low and the diffraction signal is weak. Hence detection with high sensitivity and low noise is crucial. A typical multi-wire gas detector with a research-reactor neutron source falls into this flux range, with a maximum local count rate of ∼10 × 10^3^ counts s^−1^ mm^−2^ and a global maximum count rate of ∼500 × 10^3^ counts s^−1^. However, fabrication of a large-area detector, *i.e.* a large-solid-angle detector, with this technology is difficult, and so neutron IP technology is adopted because of the very large solid angle measurement possible. Measurement methods for these area-detector-based diffractometers are similar, that is, several specimen orientations are possible and successive measurements can be made by specimen rotation in order to cover as much as possible of the reciprocal space within the available measurement time.

**Figure 3:**
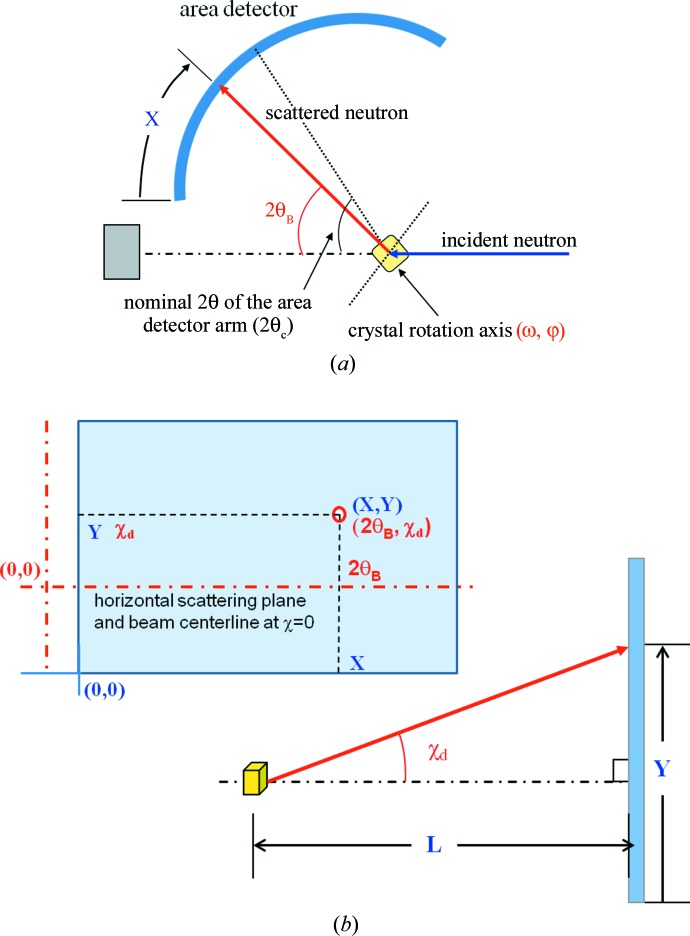
Two schematics showing the geometry of the diffractometer and its parameters for measurement. (*a*) The diffractometer configuration, similar to a two-axis diffractometer, and (*b*) a side view of the configuration from the sample to the detector, and the machine parameters (*X*, *Y*) of Bragg spots with their equivalent angular parameters (2θ_B_, χ_d_) in an image data frame (the detection area) of the area detector.

Fig. 3 ▶ shows a schematic of the C-2DPSD-based diffractometer geometry and all the parameters used in this paper. The diffraction angle in the basal plane is 2θ_B_, and two angles (ω, ϕ) for the sample rotation, *i.e.* the Ewald sphere rotation, are the same as for the four-circle diffractometer, but the angle of elevation, χ_d_, is different because the sample is only rotated by ϕ (equivalent to ω rotation with χ = 0 or a two-axis diffrac­tometer) during the measurement; a wide-area detector could measure other reciprocal lattice points off the equatorial scattering plane in the Ewald sphere.

As shown in Fig. 3 ▶(*b*), when we try to measure the diffraction signal, we get (*X*, *Y*), the position of any diffraction spot found, and the intensity at the distance from the sample position *L*. This parameter set combined with the sample rotation, ϕ, is converted to the reciprocal space point (x*, y*, z*). Here the sample is rotated around the ϕ axis, and the ω axis is always fixed with χ = 0; thus the two axes are coaxial, and the sample goniometer is used for inclination of the specimen. As the sample is rotated continuously, an image of 512 × 512 pixels is obtained at a given angular increment by Δϕ, and this image is then transferred to the computer in ∼0.3 s. As the sample rotates successively by 360°, the reciprocal lattice points limited by the Ewald sphere can be measured. It should be noted that ϕ rotation is normally a continuous scan, not a step scan. A more detailed practical consideration for the transformation to the reciprocal space point from the real space coordinates can be found in *International Tables for Crystallography*, Vol. C (Helliwell, 1999 ▶).

The Bragg spots found in these image frames are converted to the equivalent reciprocal space points, *i.e.* vectors, and then a UB matrix can be found by the vector minimum or other methods. This point will be explained in more detail in the next section. We note that the UB matrix is determined after the whole data collection measurement is finished, making the initial orientation of a specimen unimportant. This means that pre-determination of the UB matrix is not necessary for the data collection, and also all the space points covered by the scan can be searched without missing any of them.

**Figure 4:**
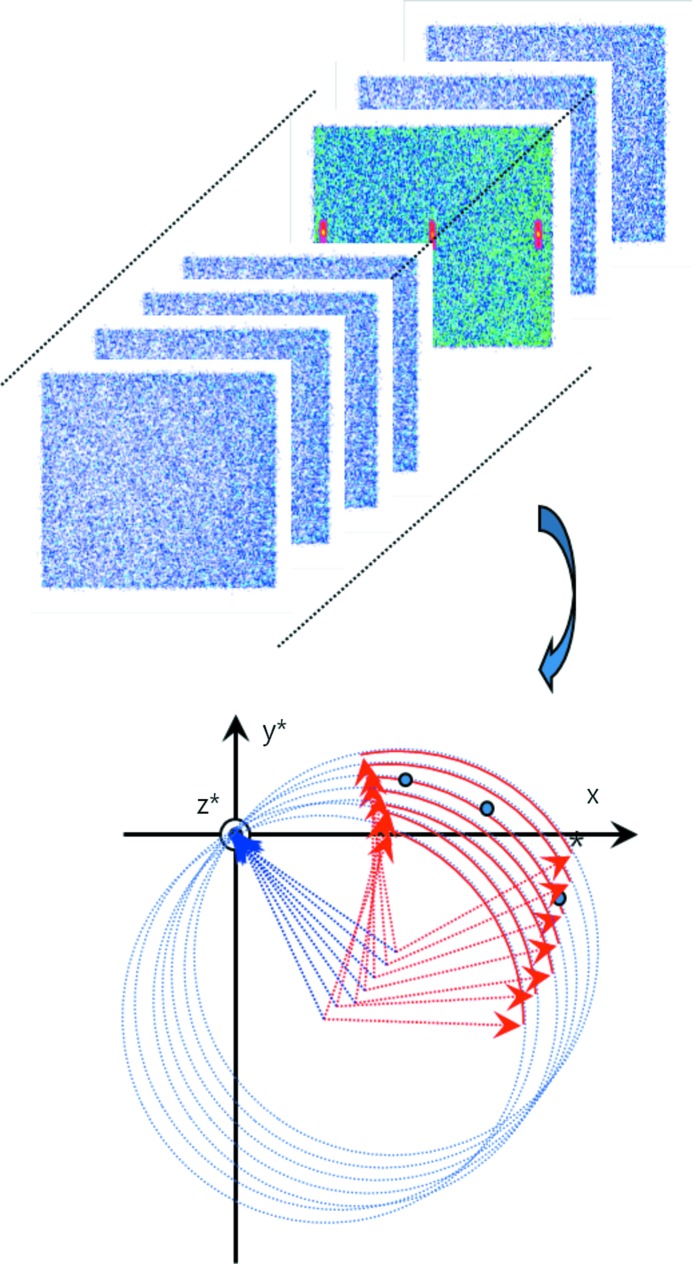
A schematic showing the measurement method by sample rotation. The sample rotates around the ϕ axis, usually by 360°, while the ω axis is fixed. When the unit step width of rotation is 0.2°, then one frame of the image data contains all the reciprocal points in that step width, Δϕ = 0.2°. The detector range in diffraction angle 2θ_B_ is 110° and so a two-step measurement is needed to cover a full diffraction range up to 160°.

With regard to the intensity measurement, the integrated intensity can be obtained by summing the intensities over peak area pixels of the Bragg spot in the image frame, as shown in Fig. 4 ▶. As stated earlier, the Bragg spot reflections have finite dispersions and so a single reciprocal lattice point appears usually over several image data frames, in which case each spot in an image frame is actually a partial portion of the reciprocal lattice point. Of course this is also dependent on the angular increment unit, Δϕ, for one frame. Therefore, the complete integrated intensity of each reciprocal lattice point should be summed over these image data frames.

### 2.3. Raw data treatment and visualization   

There are several types of single-crystal diffraction experiment and hence different requirements for each experiment. One such experiment is the collection of many Bragg reflections under given environmental conditions, such as temperature, electric or magnetic fields, pressure *etc*., for a structural solution or refinement study. This is our main target for this development. Another is the mapping or searching of a wide reciprocal space of diffuse scattering or the detection of superlattice or satellite reflections. The other widely used techniques for SCD are to measure any specified reciprocal space points, *i.e.* individual reflections in any direction (usually called a *q* scan, a generalized reciprocal space scan), and to trace reciprocal lattice points related under the environmental parameters through various phase transitions or structural changes.

In order to utilize the advantages of diffraction with a large-area detector, we need to have appropriate tools for raw data acquisition and processing to produce the required data with a convenient format for various analysis programs, together with intuitively easy, fast and functionally powerful visualization tools. One of the very different features of data produced from an area-detector diffractometer is the huge size of the raw data sets obtained compared to the case of a point-detector diffractometer. Hence the raw data processing software and the visualization tools should be able to handle these large data packets of ∼GB size. Over the past several years, we have developed and tested the modules needed to process each step from data acquisition through raw data preprocessing to data manipulation for a reflection intensity list for structure analysis; this software is now packaged into the *Reciprocal Analyzer* with a unified graphical user interface (GUI) and visualization tools (Ishikawa *et al.*, 2008 ▶; Noda *et al.*, 2011 ▶).

**Figure 5:**
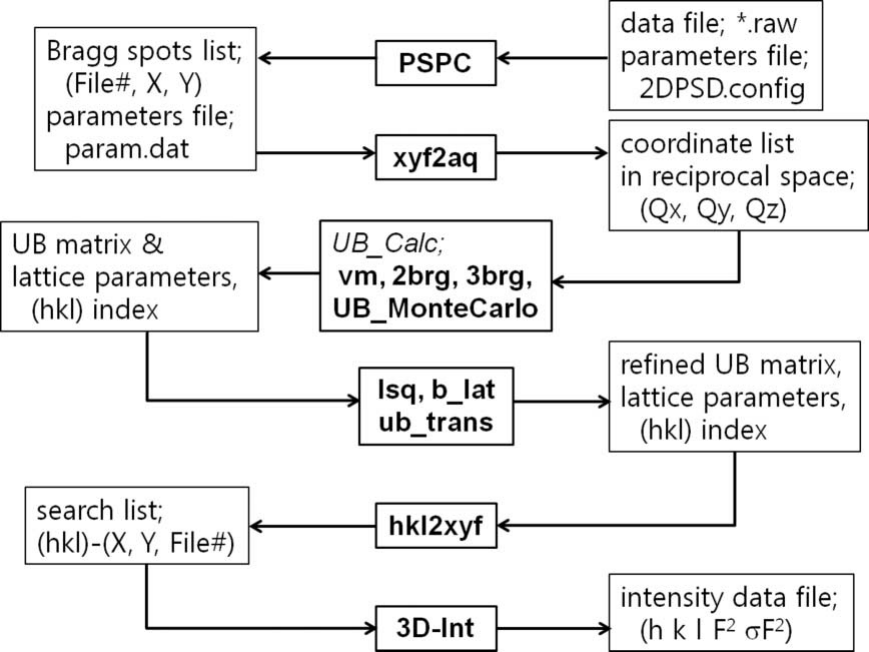
A schematic showing the flow of the raw data processing, starting from the Bragg spot search through UB matrix calculation to the production of the reflection-intensity data file for analysis by using the *Reciprocal Analyzer*.

Fig. 5 ▶ shows the overall structure and work flow in the *Reciprocal Analyzer*; the central boxes with bold characters represent each code module, *i.e.* program, and the boxes on the left and right sides are their input and output data files and parameter files. All the modules except *PSPC* and *3D-Int* are written in Fortran, and each module is linked by input–output disk files with generic names such as param.dat, ang-Q.dat, ub.dat and HKL.dat. The package is sufficiently flexible to accommodate data from various sources, including laboratory four-circle X-ray diffractometers, neutron four-circle diffractometers, two-axis X-ray diffractometers with an image plate and three-axis (2 + 1) X-ray diffractometers (except for *PSPC*, which is applicable only for the C-2DPSD diffractometer).

An image data frame is acquired and saved after an angular increment Δϕ for each frame. For example, when Δϕ is equal to 0.2°, then 1800 image frames are acquired over the complete rotation of 360°. Because there are 4 bytes per pixel in the 512 channels each in the **X** and **Y** directions, one image frame corresponds to 1 MB, and therefore the set of data amounts to 1.8 GB. When Δϕ is 0.1°, then a 3.6 GB data file is produced for a 360° measurement. The data structure of the pixel has extra information, such as the time stamp, and we could reduce the size of the data file by transforming 2 bytes only from the 4 byte format, to retain just the intensity information; in this case the maximum recorded intensity per pixel could be cut by a 16 bit number (65 535). The active working space of the image data frame does not occupy the square-shaped full channel area because the detector shape is elongated horizontally, from the angular dimensions of ∼110 × 54° of the detector. Again the net data size could be reduced physically by extracting just the active area. Finally, the reduced data file size would be ∼0.3 MB per frame, *i.e.* ∼0.54 GB per data set of 1800 frames with Δϕ = 0.2°.

The *PSPC* module, discussed below in more detail, processes a data set of image frames as a whole, together with the given parameter file, 2DPSD.config, to produce a list of peaks using the peak search routine. The module requires user inputs for the threshold level of a peak and a masking box size to select an appropriate peak area manually by eye. In the first stage, a rather higher threshold level, *i.e.* stronger peaks, is selected. The peaks list contains information in a specific format (peak number, file number, *X*, *Y*, intensity), and this information, expressed as machine parameters in real space, is converted into reciprocal space coordinates, **Q**, by the module *xyf2aq*, using the parameter file param.dat; this contains the diffractometer parameters, experiment configuration and conversion factors for those machine parameters, with control codes for program execution.

The ang-Q.dat file contains information on the observed reciprocal lattice points, including spurious peaks from various sources such as powder patterns from the sample environment. The UB matrix and indexing of each point can be obtained by applying one of the codes among *vm*, *2brg*, *3brg* and *UB_MonteCarlo*. The *vm* module produces the UB matrix from the given **Q** values of selected reflections by the vector minimum method. The *2brg* module produces the UB matrix from two selected reflections with lattice parameters, and *3brg* produces the UB matrix from three selected reflections. *UB_MonteCarlo* gives the UB matrix using the given lattice parameters. The first result might be transformed into the Bravais lattice by using *b_lat* and *ub_trans* with *lsq*, that is, the Bravais lattice finder, the UB matrix lattice transformer and the least-squares refinement module, respectively.

We now obtain a new ang-Q.dat file with the refined UB matrix and a new indexing of well defined reciprocal lattice points under the adopted Bravais lattice. If it is acceptable, a list of the complete reciprocal lattice points with their real space points (file number, *X*, *Y*) is produced by *hkl2xyf* from the UB matrix, and then *3D-Int* tries to produce the integrated intensity for each of those reciprocal lattice points over the data set of the image data frames. The final result is then produced for all the reciprocal lattice points, with detailed information on their indexing and integrated intensity corrected by the Lorentz factor (Nitta, 1961 ▶). They are also categorized by their peak quality, as ‘good peak’, ‘no peak’, ‘no good peak’, ‘peak out of expected region’ and ‘peak out of experimental area’, where ‘no good peak’ means that there is a peak intensity but it does not satisfy the criterion that the spot is not separated from the expected point by less than 0.1 reciprocal space distance, and ‘partial peak’ means that there is a peak but it resides around the boundary of the image frame and so the calculation of the integrated intensity could not be completed. From this information, the reflection-intensity list is extracted with an appropriate format, such as [*h k l F* ^2^ σ(*F* ^2^)], for analysis.

The program *DABEX* is usually applied to the reflection-intensity list for absorption correction and path length calculation, and the program *RADIEL* (Coppens *et al.*, 1979 ▶) is used for structure analysis; this program has been modified in house for improved extinction corrections. *SHELX* (Sheldrick, 2008 ▶), *FullProf* (Rodríguez-Carvajal, 1993 ▶) or *GSAS* (Larson & Von Dreele, 2000 ▶) could also be used for crystallographic or magnetic structure analysis.

**Figure 6:**
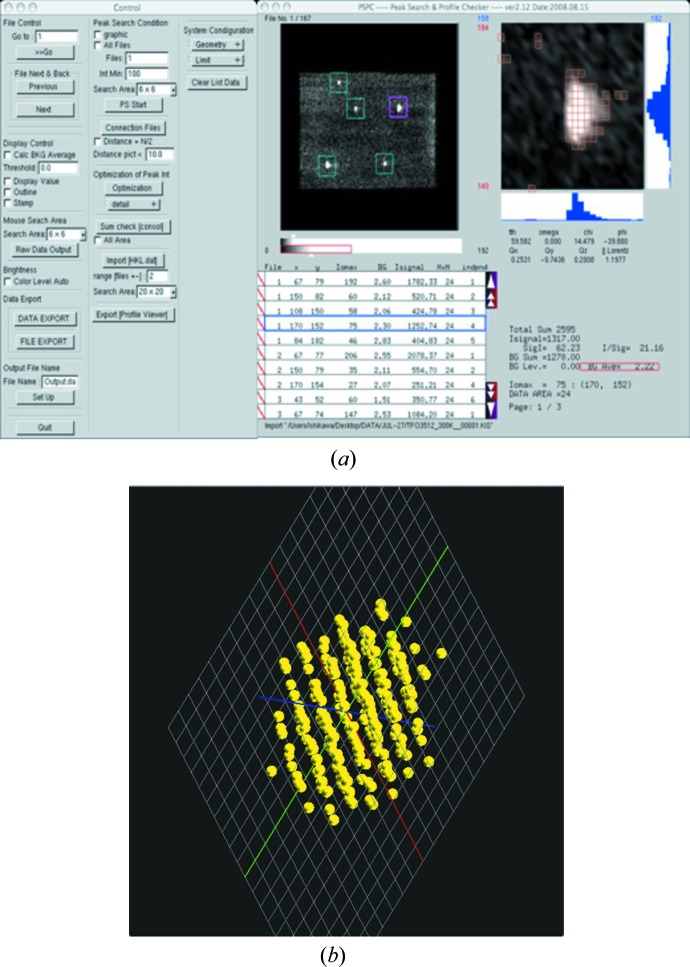
Screen capture images showing (*a*) the *PSPC* working window for the automatic peak search and (*b*) a schematic display of the spots in reciprocal space from the *Reciprocal Analyzer*.

*PSPC* is a program implementing the two-dimensional peak search function to find peaks in the image data frames under the given conditions, though it has many other functions for data treatment and crystallographic calculation, as described above [see Fig. 6 ▶(*a*) for a screenshot of its typical working screen]. Once the threshold level to judge a clear meaningful peak and the size of the square box for defining the peak area in the image frame are given by the user, then the peak search routine tries to find each peak by successively relocating the center of the search box to the center pixel calculated from the center of gravity. The integrated intensity is then calculated by successive masking for the background signal and noise to extract the net intensity. This process continues until the whole area and the last image frame have been searched. The present integration scheme assumes that each reflection can be isolated within the box. When the first peak search process is finished, the user can visually inspect all the peaks that were found automatically by the program, as shown in Fig. 6 ▶(*a*), and add or remove possible peaks manually. The second process then combines those distributed peaks over several consecutive image frames.

There are two visual display sub-windows in *PSPC*, as shown in Fig. 6 ▶(*a*), and one separate window, as shown in Fig. 6 ▶(*b*), called the reciprocal space viewer in the *Reciprocal Analyzer*. The left sub-window in *PSPC* represents each image frame display as a whole, showing the status of peaks found, and the right sub-window shows a single peak covered by the search box with histograms in the horizontal and vertical directions. Visualization requires clarity and speed without undue loss of information, and the ability to guide the experimenter by providing necessary information in the bottom part of the GUI. The reciprocal space viewer shown in Fig. 6 ▶(*b*) displays the three coordinate axes of the reciprocal space under consideration and the lattice points found by *PSPC* in three dimensions; the viewer can be used to show various situations of the measured reciprocal lattice point distribution by manipulating flags in the reflections list in order to change the colors on the screen and the rotation of axes in any direction.

The *PSPC* program, the visual display modules and the packaging code integrating all the modules in the *Reciprocal Analyzer* are written in the C programming language with the OpenGL library and GUI toolkit. They can therefore be easily ported to different OS platforms, and actually three versions of the code are available, on Windows, Mac and Linux, to satisfy the demands of the diverse user base in a neutron scattering laboratory.

### 2.4. Calibration of the diffractometer using an NaCl single crystal   

The calibration of a diffractometer is usually carried out using an NaCl single crystal, a *de facto* laboratory standard specimen, by refining the various conversion factors, distances, zero offsets, wavelengths and standard detector angles.

**Figure 7:**
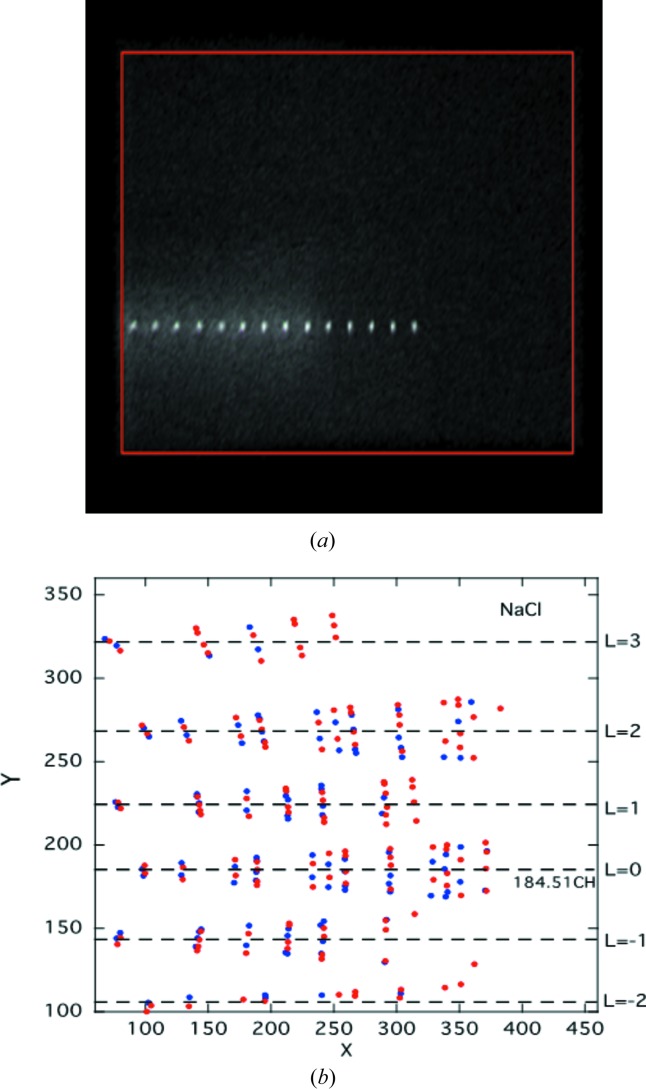
(*a*) Typical image data for the conversion factor in 2θ and Δ*X* during machine parameter calibration using NaCl 220 Bragg spots and (*b*) schematic plots of NaCl Bragg spots in (*X*, *Y*) space collected using two detector arm positions.

First, the conversion factor (Δ2θ_B_/Δ*X*) for angle 2θ_B_ from the *X*-channel coordinate is obtained by measuring diffraction spots; an *hk*0 reflection in the equatorial scattering plane is measured as a function of the mechanical step movement of the detector unit itself, *i.e.* 2θ, as shown in Fig. 7 ▶(*a*), in which the NaCl 220 reflection is measured by moving the detector with a step of 5°. All the diffraction spots found by *PSPC* from a complete scan over 360° rotation of the NaCl crystal are plotted in Fig. 7 ▶(*b*), which clearly shows the group in *l* layers of reflections. By averaging *h* *k*0 and 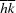
0, we can obtain the distance (Δ*Y*) between the *l* = 0 and *l* = 1 layers. Then using these reflections, the equator line information *Y*_0_, the detector arm nominal position *X*_0_ at 2θ_c_ and the slope of the *X*–*Y* channels could be obtained. With regard to the conversion factor for the angle of elevation, χ_d_, from these reflections in the *Y* channels, we use the conversion factor, Δχ_d_/Δ*Y*/*L*.

Next, the wavelength λ of the neutron beam from each monochromator position for different wavelengths should be calibrated. This can be done by plotting *hk*0 reflections in the horizontal scattering plane against the *X* channel and least-squares fitting, assuming the given lattice parameter of the NaCl crystal, using the Bragg equation. When another wavelength is selected or any working condition of the diffractometer components is changed, the calibration procedure described above should be repeated. Typical results of the calibration are summarized in Table 2 ▶. By carrying out this calibration process precisely, any misalignment of the monochromator, spectrometer table or detector unit can also be detected, and this process therefore should be done regularly at the beginning of each reactor operation cycle.

## 3. Applications   

### 3.1. Large-single-crystal check   

One of the fastest and most direct applications is a crystal quality and orientation check, which is especially useful for large-volume crystals using the high penetration power of neutrons. As a typical example, a YMn_2_O_5_ single crystal of ∼1 × 1 × 1 cm could be measured in ∼50 min to obtain the UB matrix in addition to performing a quality check. This kind of measurement can be done usually within 1 h for widely varying conditions in volume, crystal system and shape to obtain information on the crystallinity, twins, daughter crystallites and so on.

### 3.2. NaCl structure analysis   

The first trial structure analysis was conducted on NaCl. An NaCl single crystal with dimensions of 2 × 2 × 2 mm was measured by ϕ rotation, over two nominal 2θ positions of the detector arm, 2θ_c_ = 40 and 80°, to cover a full diffraction angle range of up to 160°. As described in §[Sec sec2.3]2.3, the reflection-intensity list was extracted. Then the program *DABEX* was applied to *F* ^2^ and s.u. σ(*F* ^2^) for the absorption correction and path length calculation. This corrected reflection-intensity list was input into the program *RADIEL* to obtain the structural parameters with extinction corrections.

**Figure 8:**
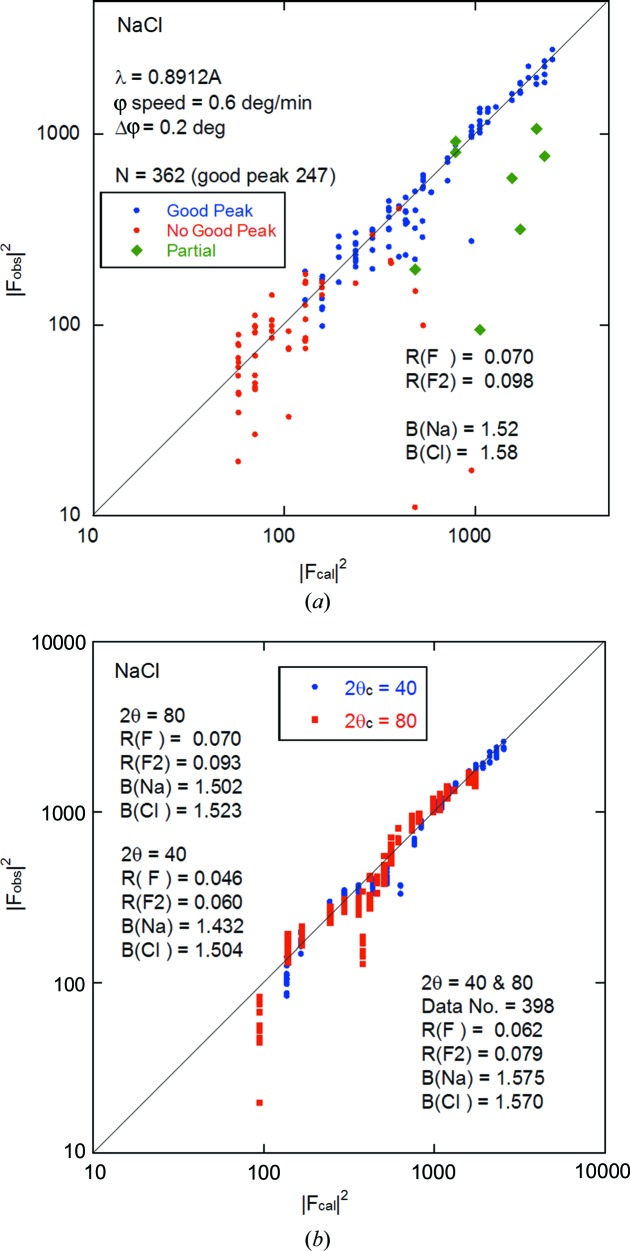
Results of structure analysis on NaCl from the C-2DPSD diffractometer at different wavelengths. (*a*) An *F*_obs_^2^ *versus* *F*_cal_^2^ plot from the measurement at the neutron wavelength 0.891 (1) Å, and (*b*) the corresponding plot at 1.153 (1) Å. The designations 2θ = 40 and 80° represent two measurement arrangements of the detector denoted by its arm angle reading. The vertical and horizontal axes are in logarithmic scale.

Fig. 8 ▶(*a*) shows a plot of all of the 391 reflections found, namely 247 good peaks and 144 ‘no good’ and partial peaks, taken at a detector nominal position of 40°. Because the wavelength was as short as 0.8912 Å, this measurement could capture the reflections of the *l* = −2 to *l* = +3 layers (see Fig. 7 ▶ *b*), but the spots appearing at *l* = −2 gave a lot of partial peaks owing to the location around the image boundary, and they were removed for analysis as partial peaks. The reliability factors are given as *R*(*F*) = 0.070 and *R*(*F* ^2^) = 0.098, respectively, with isotropic atomic displacement parameters of *B*(Na) = 1.52 and *B*(Cl) = 1.58. The unit-cell parameters for the 2θ_c_ = 40° measurement were *a* = 5.658 (3), *b* = 5.657 (2), *c* = 5.655 (6) Å, α = 89.97 (7), β = 89.99 (7), γ = 89.98 (4)°.

When measured at a neutron wavelength of 1.3143 Å, as shown in Fig. 8 ▶(*b*) (taken at two detector nominal positions of 40 and 80°, and analyzing the data together), reliability factors *R*(*F*) = 0.062 and *R*(*F* ^2^) = 0.075, and isotropic atomic displacement parameters of *B*(Na) = 1.575 and *B*(Cl) = 1.570 with 398 good reflections were obtained. If we analyzed the data taken at the two positions separately, their *R*(*F*) and *R*(*F* ^2^) factors were 0.046 and 0.060 for 40° data, and 0.070 and 0.093 for 80° data, respectively. Thus the reliability and reproducibility proved to be satisfactory.

### 3.3. Structure analysis of Tb_3_Fe_5_O_12_   

Terbium iron garnet (TbIG), Tb_3_Fe_5_O_12_, is known to have a normal cubic crystal structure at room temperature, with space group 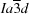, where the Wyckoff positions are Tb 24*c*, Fe 16*a*, Fe 24*d* and O 96*h*, and a lattice constant of 12.4364 Å. A preliminary structural study was carried out on a TbIG single crystal with and without a magnetic field of 2 kG along the cube diagonal direction [111].

**Figure 9:**
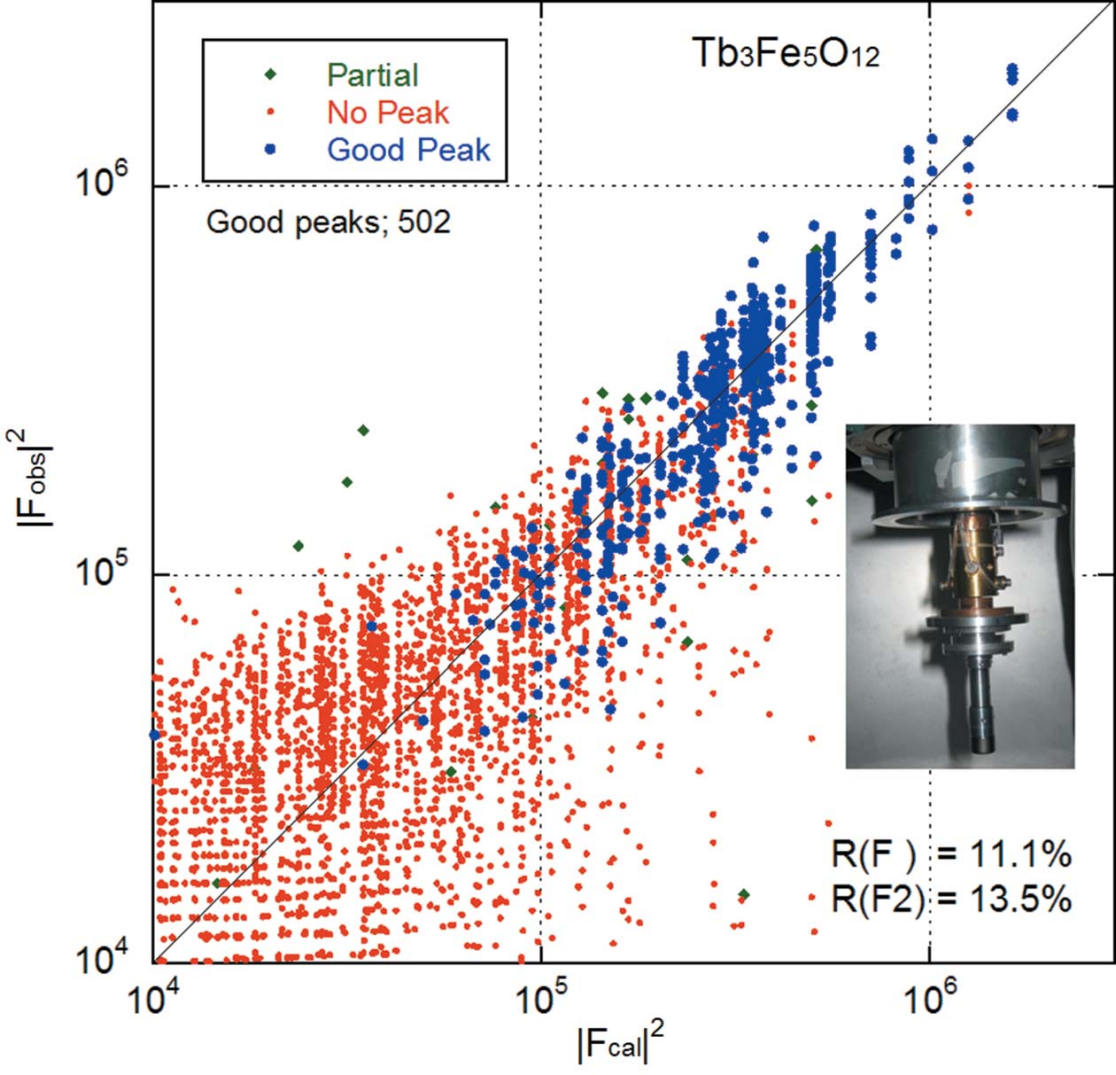
Structure analysis results of terbium iron garnet, Tb_3_Fe_5_O_12_, at room temperature with a magnetic field of 2 kG for 502 good peaks. The inset photograph shows the magnetic field device attached to the cold head. The vertical and horizontal axes are in logarithmic scale.

Fig. 9 ▶ shows the measurement results taken over a 360° full sample rotation with a magnetic field of 2 kG at room temperature, and the inset photograph in the figure shows the permanent magnetic field device used, attached to the low-temperature sample environment. The measurement yielded 503 good peaks and 42 partial peaks out of a possible 8121 peaks, and resulted in *R*(*F* ^2^) = 0.1354 and lattice parameters of *a* = 12.487 (6), *b* = 12.480 (6), *c* = 12.474 (6) Å, α = 89.97 (4), β = 90.04 (4), γ = 89.97 (4)°. Low-temperature measurements with the magnetic field along the [111] direction and a magnetic structure analysis will be published elsewhere.

### 3.4. Composition analysis of a mixed crystal of (Tm_*x*_Yb_1−*x*_)Mn_2_O_5_   

**Figure 10:**
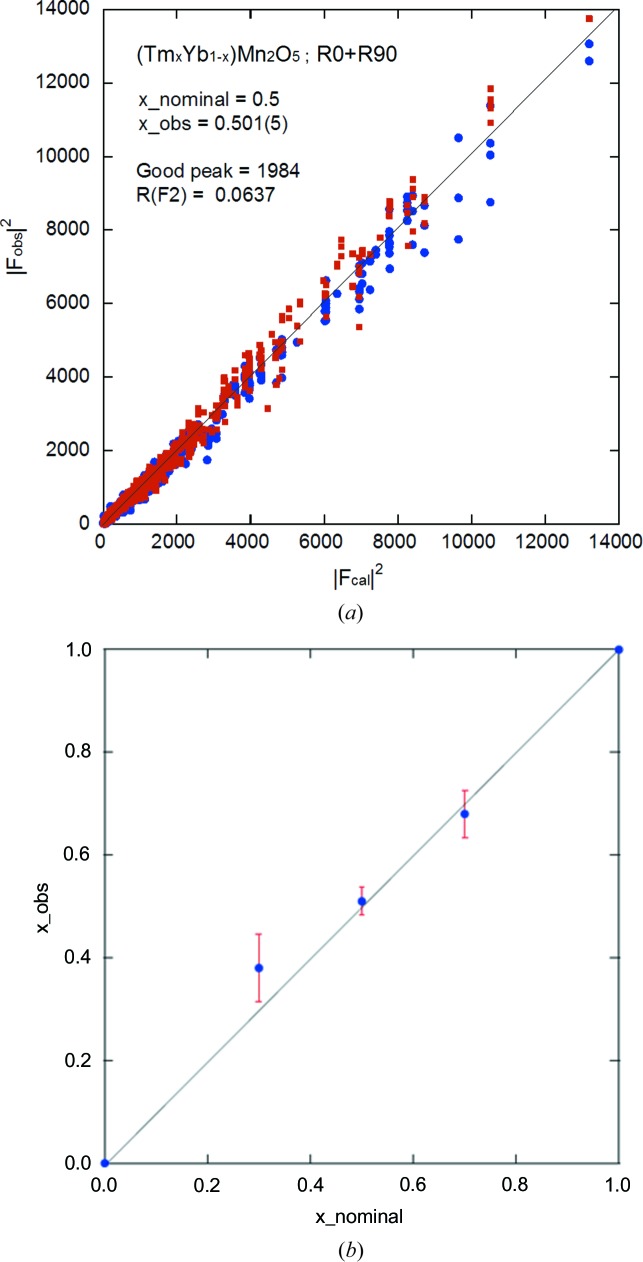
Analysis results for a (Tm_*x*_Yb_1−*x*_)Mn_2_O_5_ single crystal. (*a*) *F*_obs_^2^ *versus* *F*_cal_^2^ plot (R0 and R90 represent the sample rotated orientation with respect to the sample goniometer vertical axis) and (*b*) a plot showing the element composition of different crystals of (Tm_*x*_Yb_1−*x*_)Mn_2_O_5_ and their structural configuration depending on the replacement of elements.

One convenient and powerful application is the rapid determination of replacement element or dopant composition in various compounds or solid solutions, as shown in Fig. 10 ▶(*b*). Fig. 10 ▶(*a*) shows one example of such an analysis, wherein a series of (Tm_*x*_Yb_1−*x*_)Mn_2_O_5_ single crystals were subjected to half to one day measurements to analyze the effect of replacing an element just after growth, as shown in Fig. 10 ▶(*b*). The oxygen position distortion, depending on the composition, was also found to be consistent with high precision and accuracy. For the (Tm_*x*_Yb_1−*x*_)Mn_2_O_5_ case, X-rays cannot distinguish between the elements Tm and Yb, but neutrons can do this very easily. This result with other physical measurements will be published elsewhere.

### 3.5. Hydrogen-containing materials   

Neutrons have unique scattering cross sections for many elements, and one typical case would be hydrogen, where the cross sections of hydrogen and deuterium are significantly different: a large incoherent cross section and negative coherent cross section for hydrogen, and an appreciable positive coherent cross section value, similar to that for carbon, for deuterium. Single-crystal neutron diffraction is less disturbed by the incoherent scattering cross section of hydrogen. In order to demonstrate the advantage of neutron diffraction for the structural study of materials containing hydrogen with fairly large unit cell volume, the compound taurine was grown and characterized by a laboratory X-ray FCD and the C-2DPSD diffractometer.

**Figure 11:**
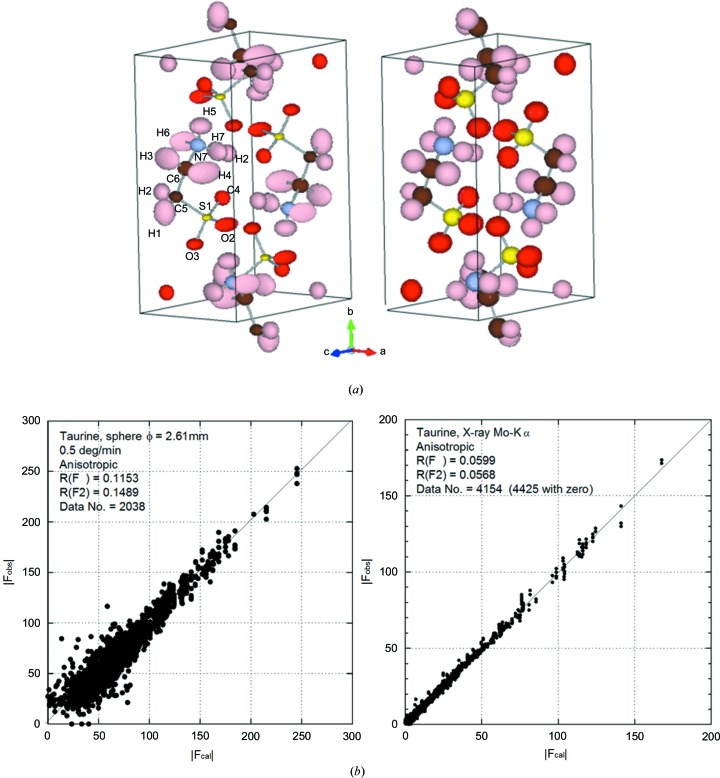
Structure analysis results for the organic compound taurine. (*a*) A ball-and-stick model of the taurine specimen with anisotropic atomic displacement parameters for H atoms, obtained by neutron diffraction results from the C-2DPSD diffractometer, and the equivalent model obtained using results from an X-ray four-circle diffractometer with isotropic atomic displacement parameters for H atoms (one H atom was fixed on the basis of the neutron data). (*b*) A comparison of *F*_obs_ and *F*_cal_ for the neutron and X-ray structure analysis.

X-ray diffraction measurements on a specimen of 0.6 mm diameter using an Mo target gave the result as a primitive monoclinic crystal system, space group *P*2_1_/*c*, with lattice parameters *a* = 5.2813 (2), *b* = 11.6416 (4), *c* = 7.9203 (3) Å, α = 90.001 (3), β = 94.093 (3), γ = 89.997 (3)°, and a unit-cell volume of 485.72 (3) Å^3^. The structural analysis results are shown on the right side of Figs. 11 ▶(*a*) and 11 ▶(*b*). Some of the H atoms were fixed on the basis of the following neutron structure analysis.

The specimen for neutron diffraction was a sphere-shaped crystal with 2.61 mm diameter. Neutron diffraction with a wavelength of 1.153 Å was measured using conditions of 0.5° min^−1^ rotation, 0.2° per frame over 360° in ϕ (12 h net measurement time), and two nominal 2θ counter arm positions equal to 37 and 80° with two specimen orientations of the sample rod of 0 and 90°, respectively, and with tilting of the crystal of about 30° using the sample goniometer head. This produced a total of four sets of 1800 image data frames. The analysis results from 2038 reflections could be summarized by the lattice parameters *a* = 5.296 (3), *b* = 11.680 (10), *c* = 7.947 (6) Å, α = 90.01 (7), β = 94.16 (5), γ = 89.97 (6)° with a unit-cell volume of 490.3(6) Å^3^ and are shown on the left side of Figs. 11 ▶(*a*) and 11 ▶(*b*). The ball and stick models in Fig. 11 ▶(*a*) were produced by the program *VESTA* (Momma & Izumi, 2011 ▶).

### 3.6. Diffraction study trial on bio-materials   

One of the objectives of the development project of the C-2DPSD-based diffractometer is to build the capability to study large-unit-cell-volume systems of around ∼10 000 Å^3^ or greater, such as macromolecular and bio-related materials. The material Na_2_UMP·7H_2_O (UMP is uridine monophos­phate) was used to measure diffraction of 2.204 Å neutrons at a speed of rotation of 0.1° min^−1^ over 360°, that is, ∼2.5 days of measurement time per 1800 image data frames. The material Na_2_UMP·7H_2_O has atomic element numbers Na_48_C_216_N_48_P_24_O_384_H_600_ in the unit cell, with the ortho­rhombic system *P*2_1_2_1_2_1_. The trial measurement produced 1547 Bragg spots among 7625 possible Bragg positions, with unit-cell lattice parameters *a* = 8.89 (2), *b* = 23.04 (2), *c* = 58.72 (4) Å, α = 90.04 (6), β = 89.94 (12), γ = 90.00 (17)° and volume 12 025 (31) Å^3^. Preliminary analysis of this limited number of measured reflections showed that the structure could be solved, including the H atoms, when all available reciprocal lattice points were measured.

## 4. Concluding remarks   

A new neutron single-crystal diffractometer has been developed, based on a large-area curved position-sensitive detector covering a range of 2θ_B_ = 110°, χ_d_ = 54°, equipped with a Ge(311) mosaic monochromator and supermirror vacuum beam paths. We have developed a measurement methodology as well as an integrated software package, the *Reciprocal Analyzer*, for processing raw data sets with a size of the order of GB in order to find the UB matrix, index reflections and list reflection intensity as well as to make corrections for structural analysis. During this process, flexible tools for visualization of reciprocal space point distribution and diffraction spots in the raw data image frames have been provided, closely linked together *via* software modules in the code package. Along with this raw data preprocessing software development, a precise calibration method and procedure for determining the diffractometer machine parameters were also devised and refined using an NaCl single crystal. Commissioning has been completed as described and thereby the instrument milestone of ‘launching for users’ has now been reached.

The task of correction and calibration using an NaCl crystal would be the actual starting point for various applications for this diffractometer. We have demonstrated applications such as crystallographic and magnetic structure measurements using NaCl and Tb_3_Fe_5_O_12_ single crystals, a rapid and direct crystallinity check on large crystals of YMn_2_O_5_ of ∼1 × 1 × 1 cm, and the study of the composition or dopant content of diverse compounds using a series of (Tm_*x*_Yb_1−*x*_)Mn_2_O_5_ single crystals. We demonstrated the unique scattering power of neutrons in hydrogen-containing materials using taurine single crystals compared with typical X-ray results, and showed a promising extension to large-unit-cell systems of ∼10 000 Å^3^ using an Na_2_UMP·7H_2_O single crystal. The applications could be extended to diffuse scattering measurements and industry-based texture measurements.

## Figures and Tables

**Table 1:** Possible wavelength selection of the monochromator

Angle position ()	Reflection plane	()
9.46	Ge(511)	0.736
0	Ge(311)	1.153
+10.09	Ge(422)	0.780
+29.57	Ge(111)	2.204

**Table 2:** Summary of the typical machine parameters

Conversion factor in 2_B_/*X*	0.2614 (1) per channel
Conversion factor in _d_/*L*/*Y*	0.003917 per channel
*X* *Y* slope	0.0041
Origin point (*X*_0_, *Y*_0_) in channels	(181.50, 94.135)
Active range in (*X*, *Y*) in channels	(60, 100)(460, 360)
2_B_ range at the detector arm position 40	7.8112.2
_d_ range	18.434.5
